# Association of Galectin-9 Soluble Immune Checkpoint with Clinical Prognostic Markers in Patients with Chronic Lymphocytic Leukemia

**DOI:** 10.3390/ijms27010098

**Published:** 2025-12-22

**Authors:** Aviwe Ntsethe, Phiwayinkosi Vusi Dludla, Bongani Brian Nkambule

**Affiliations:** 1 Department of Human Physiology, Nelson Mandela University, Gqeberha 6031, South Africa; 2 School of Medicine, College of Health Sciences, University of KwaZulu-Natal, Durban 4000, South Africa; 3 Department of Biochemistry and Microbiology, University of Zululand, KwaDlangezwa 3880, South Africa

**Keywords:** chronic lymphocytic leukemia, galectin-9, soluble immune checkpoints, beta-2 microglobulin, Rai stage, prognostic markers

## Abstract

Chronic lymphocytic leukemia (CLL) is a heterogenous disease, with varied clinical outcomes. Multiplex assays used to measure soluble immune checkpoints offer a less laborious method of monitoring patients with CLL, but none of these panels have been validated. The aim of the study was to assess soluble immune checkpoint profiles in patients with CLL and to correlate these with independent prognostic markers such as β2-microglobulin (B2M), Rai stage, fluorescence in situ hybridization (FISH) status, and the International Prognostic Index for Chronic Lymphocytic Leukemia (CLL-IPI). We measured plasma levels of soluble interleukin-2 receptor alpha (sCD25), T cell immunoglobulin and mucin domain-containing protein 3 (TIM-3), galectin-9, programmed cell death 1 (PD-1), programmed death-ligand 1 (PD-L1), and cytotoxic T-lymphocyte associated protein 4 (CTLA-4) using cytometric bead array-based assays. We further measured plasma levels of B2M using an enzyme-linked immunosorbent assay (ELISA) kit. Soluble immune checkpoints were correlated with prognostic markers. The plasma levels of sCD25, TIM-3, galectin-9, PD-1, and PD-L1 were significantly increased in patients with CLL compared to the control group, *p* < 0.0001. Galectin-9 plasma levels were directly associated with B2M levels (β = 0.65, *p* = 0.012). Our findings suggest that galectin-9 may provide valuable prognostic significance for patients with CLL.

## 1. Introduction

Chronic lymphocytic leukemia (CLL) is a heterogeneous disease with a variable clinical course [1]. Not all patients with CLL require treatment; in fact, many live for years without the need for therapy [2]. Some patients may present with an aggressive disease necessitating early treatment, while others have a more indolent course and may never need treatment [3]. The heterogeneous clinical course of CLL makes it essential to stratify patients into appropriate risk groups to prioritize treatment [1]. Currently, the most robust prognostic marker in patients with CLL is the mutational status of the immunoglobulin heavy-chain variable region (IGHV), which is a valuable in predicting the clinical outcomes at diagnosis [4]. Mutated IGHV is associated with slower disease progression, improved overall survival rate, and a less advanced Binet stage [5]. However, routine IGHV testing remains inaccessible in many low-resource settings, including several parts of Africa, due to cost and technical requirements [6].

Chromosomal abnormalities detected via fluorescence in situ hybridization (FISH) and metaphase cytogenetic testing offer vital prognostic insights for patients with CLL, which assist in the prediction of survival outcomes and disease progression [7]. Determining the FISH status at diagnosis is recommended for all patients with CLL and is valuable for monitoring the disease [8]. Based on chromosomal aberrations, patients can be stratified into four prognostic subgroups, ranked from highest to lowest risk: del(17p), del(11q), trisomy 12, and del(13q) [9]. Several studies have shown that patients with del(17p) have poorer outcomes when treated with combination chemoimmunotherapy [10,11]. Notably, ibrutinib-treated patients with del(17p) show worse survival rates compared to those with del(11q) or without either abnormality [12]. Interestingly, there is an apparent higher prevalence of del(17p) among Africans with CLL [13].

Elevated levels of galectin-9, released primarily by leukemic cells and cells within the tumor microenvironment have been observed in patients with CLL, and correlate with poor prognosis [14,15]. The signaling pathway involving galectin-9 and T cell immunoglobulin and mucin-domain-containing-3 (TIM-3) play a crucial regulatory role in CLL [15]. In fact, an in vitro study demonstrated that inhibiting the galectin-9/TIM-3 signaling pathway in CLL partially restores the T cell subset balance [16]. Increased expression of several inhibitory receptors such as TIM-3, programmed death-1 (PD-1), programmed death-ligand-1 (PD-L1), and cytotoxic T lymphocyte associated protein-4 (CTLA-4) modulate immunosuppression in terms of cytokine production [17]. The role of PD-1 in influencing the activity and progression of CLL has been hypothesized, with interactions mediated by PD-L1 playing a crucial role in the regulation of cytokine production [18,19]. Altered levels of CTLA-4 have also been reported in patients with CLL; however, these differ among patients [20]. While these markers are known to modulate cytokine production and immune suppression, their expression patterns differ markedly between cell-associated and soluble forms. These soluble immune checkpoints offer a less laborious method of monitoring patients with CLL, but none of these panels have been validated.

The treatment landscape for CLL has advanced significantly in recent years with the introduction of targeted therapies, ushering in a chemotherapy-free era of CLL treatment. The evolving therapeutic landscape, dominated by Bruton tyrosine kinase inhibitors (BTKis) and B-cell lymphoma 2 (BCL2) inhibitors, has further emphasized the need for biomarkers that are accessible and clinically informative, particularly in regions where genomic testing is limited [21]. The incorporation of various prognostic markers for personalized patient assessment remains a challenge. Despite growing interest in immune checkpoints in CLL, there is limited evidence describing soluble immune checkpoint profiles in African patients specifically. Existing studies were largely conducted in European or North American cohorts, despite known differences in cytogenetic profiles among African populations. This represents a critical knowledge gap, as the generalizability of existing immune-based prognostic frameworks to African patients remains uncertain.

Furthermore, although multiplex assays such as the LEGENDplex™ HU Immune Checkpoint Panel 1-S/P (10-plex) system offer the ability to measure multiple soluble immune checkpoints simultaneously, there is little guidance on which combinations provide the most clinically relevant information. For this study, a targeted 6-plex panel was selected based on prior evidence implicating these six molecules in CLL immune dysregulation. Therefore, the aim of the study was to assess soluble immune checkpoint profiles in a cohort of African patients with CLL and to further correlate them with independent prognostic markers and the International Prognostic Index for Chronic Lymphocytic Leukemia (CLL-IPI).

## 2. Results

### 2.1. Patient Characteristics and Hematological Parameters

This study consisted of 33 participants in total, comprising 21 untreated patients with CLL and 12 healthy controls. The included participants were of African *(n* = 30), European (*n* = 2), and Indian (*n* = 1) ancestry. The gender distribution was 39.39% females and 60.61% males (Table 1). None of the patients in this study were receiving treatment at the time of sample collection. The baseline characteristics and hematological parameters (Table 1) have been previously described [22]. Briefly, the mean age among patients with CLL was 62.33 ± 13.31 years, and 56.58 ± 15.67 years for the healthy controls. Furthermore, the white blood cell count in CLL patients was significantly elevated (130.4 × 10^3^ ± 29.71) when compared to healthy controls (5.26 × 10^3^ ± 1.38), *p* = 0.0005. The red blood cell count and hemoglobin level were notably lower in CLL patients when compared to healthy controls (*p* < 0.0001). No statistically significant differences were observed in platelet counts between patients with CLL and healthy controls (*p* = 0.1831) as previously described [22,23].

### 2.2. Clinical Staging and Prognostic Markers in Patients with CLL

The Rai classification system [24] was used to stage patients with CLL, and patients with CLL were classified as follows: 47.61% were classified as stage IV, 28.60% as stage III, and 23.81% as stage II (Table 2). With respect to cytogenetic profiles, the most frequent abnormality detected was deletion 11q22 (33.33%). This was followed by deletion 13q14 (28.60%) and deletion 17p13 (14.30%). Trisomy 12 was identified in one patient (4.80%), while 19% of patients showed no detectable cytogenetic aberrations (Table 2). A detailed overview of the biological and molecular features for individual patients is provided in the Supplementary Material (Table S1).

### 2.3. Elevated Soluble Immune Checkpoint Levels in Patients with CLL

**Figure 1:**
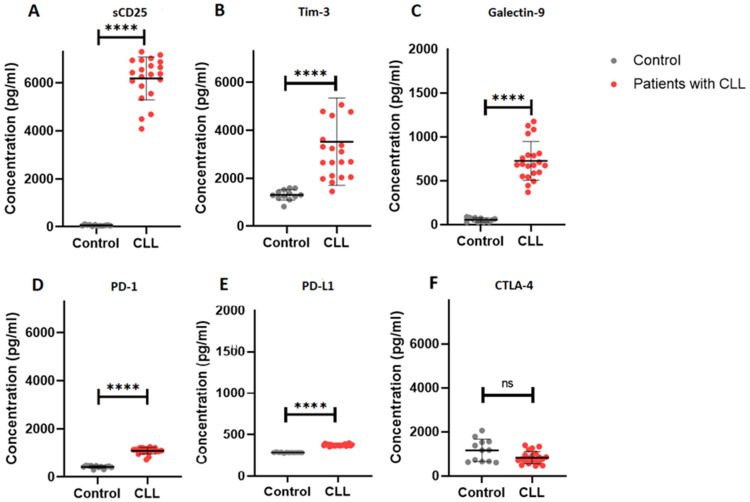
Soluble immune checkpoint profiles in patients with CLL. (**A**–**F**) illustrates the concentration (pg/mL) of sCD25, TIM-3, galectin-9, PD-1, PD-L1 and CTLA-4, respectively. The data is presented as the mean ± standard deviation (SD). **** *p* < 0.0001, ns = not significant.

We evaluated the levels of the soluble immune checkpoints in patients with CLL (Figure 1). There was a significant increase in the levels of sCD25 (IL-2Ra) in patients with CLL (6183 ± 893.40) compared to the control group (59.99 ± 23.87), *p* < 0.0001. In addition, patients with CLL had higher TIM-3 levels (3521 ± 1817) when compared to the control group (1304 ± 217.8), *p* < 0.0001. Furthermore, galectin-9 levels were significantly increased in patients with CLL (725.9 ± 221.5) when compared to the control group (53.42 ± 23.55), *p* < 0.0001.

PD-1 levels were significantly elevated in patients with CLL (1088 ± 129.7) compared to controls (417.8 ± 57.05), *p* < 0.0001. Similarly, PD-L1 levels in patients with CLL were significantly increased (373.4 ± 11.18) in comparison to the control group (284.0 ± 3.689), *p* < 0.0001. However, CTLA-4 levels were comparable between patients with CLL (832.5 ± 276.1) and the control group (1161 ± 503.3), *p* = 0.0542.

### 2.4. Association of Galectin-9 with β2 Microglobulin in Patients with CLL

**Figure 2:**
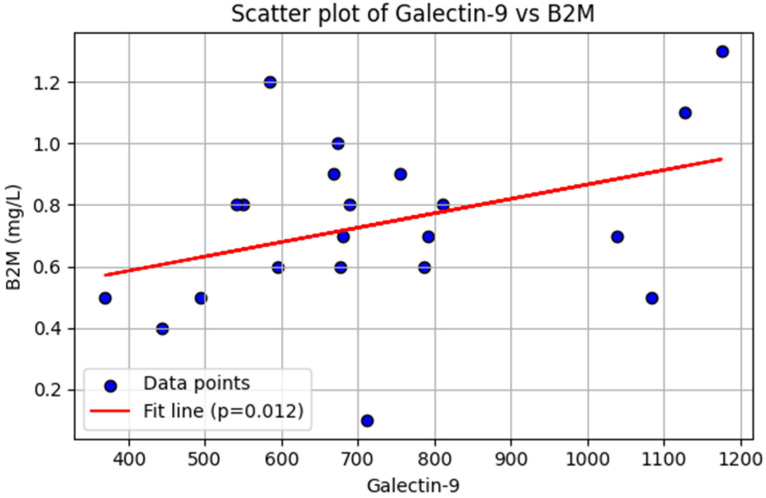
Linear regression analysis. Association between galectin-9 and β2-microglobulin (B2M) levels in patients with chronic lymphocytic leukemia (*n* = 21). Each dot represents an individual patient. The red line indicates the fitted regression line, demonstrating a significant positive association (*p* = 0.012).

In our multivariable regression model, adjusted for age and sex, there was no association between B2M levels and sCD25 levels (β = −0.048, *p* = 0.971), TIM-3 levels (β = −3.00, *p* = 0.196), PD-1 levels (β = 0.25, *p* = 0.123), or PD-L1 levels (β = −0.0065, *p* = 0.733) in patients with CLL (Supplementary Table S1). However, B2M levels were positively associated with galectin-9 levels (β = 0.65, *p* = 0.012) in patients with CLL (Figure 2).The Rai stage showed no statistically significant association with sCD25 levels (β = −901.67, *p* = 0.274), TIM-3 levels (β = −2521.88, *p* = 0.078), galectin-9 levels (β = 218.93, *p* = 0.099), PD-1 (β = 56.00, *p* = 0.537) levels, or PD-L1 levels (β = 2.21, *p* = 0.843) (Supplementary Table S2).

In a logistic regression model, no statistically significant association was observed between soluble immune checkpoints and FISH profiles or CLL-IPI score (Supplementary Table S3).

## 3. Discussion

The aim of this study was to assess soluble immune checkpoint profiles in patients with CLL and to correlate them with independent prognostic markers and the International Prognostic Index for Chronic Lymphocytic Leukemia (CLL-IPI). In our study, we found elevated levels of the soluble immune checkpoints galectin-9, TIM-3, CD25, PD-1, and PD-L1 in patients with CLL (Figure 1). In malignancies, elevated levels of immune checkpoints are associated with T cell exhaustion and may be indicative of poor prognosis [25,26].

The hematological characteristics of the patients in our cohort demonstrate elevated average white blood cell (WBC) count despite 76.2% patients being on “watch and wait” at the time of sample collection. This may be attributable to the fact that approximately 50% of patients are at the most advanced stage of the Rai staging system. Therefore, more than half of patients in our cohort may have characteristics of splenomegaly and enlarged lymph nodes. These clinical parameters may explain the high levels of plasma immune checkpoints in our cohort. However, due to the limited sample size of the cohort, the relationship between these clinical parameters and soluble immune checkpoints has not been explored.

TIM-3 is an inhibitory protein that interacts with galectin-9 and inhibits T cell activation to regulate immune tolerance [27]. In patients with CLL, elevated levels of galectin-9 stimulate the proliferation and activation of regulatory T cells (Tregs) which suppress the function of helper T cells [16]. In this study, we report on increased levels of both soluble TIM-3 and galectin-9 in patients with CLL. Elevated levels of galectin-9 and TIM-3 in patients with CLL may suggest a dysregulation of the immune system and possibly an unfavorable disease course. These immune checkpoint molecules contribute to immune evasion by malignant cells, promoting tumor cell survival and growth [25,26]. The interaction between galectin-9 and TIM-3 promotes immune suppression by inhibiting the activation of helper T cells, thereby exacerbating the proliferation of malignant cells [16].

Our findings are consistent with those reported by Taghiloo et al., who reported on elevated galectin-9 levels in patients with advanced-stage CLL [28]. Taken together, these findings suggest that galectin-9 could serve as a potential indicator of poor prognosis. Moreover, a recent study found that both galectin-9 and TIM-3 were elevated in the regulatory T cells of patients with CLL in the Binet C stage [16], indicating that the TIM-3/galectin-9 interaction may be associated with poor prognosis in patients with CLL. The TIM-3/galectin-9 axis exerts its function by stimulating regulatory T cell differentiation and inhibits Th17 cell differentiation [29]. In a recent study, the inhibition of the TIM-3/galectin-9 pathway suppressed the function of regulatory T cells, suggesting that this pathway could be a novel target for immunotherapy in patients with CLL [16].

In addition to galectin-9’s immunosuppressive role, our study found that galectin-9 levels positively correlated with B2M, a well-established independent prognostic marker in CLL. Given its association with both advanced clinical stages and immune dysregulation, galectin-9 may serve as a biomarker of poor prognosis in CLL. Similar findings were reported by Ahmed et al., who demonstrated significantly higher galectin-9 serum levels strongly associated with B2M and advanced clinical stages [30]. Notably, other soluble immune checkpoints in our panel did not show significant correlations with B2M. This may reflect fundamental biological differences in their cellular sources and functional roles in CLL. Moreover, the absence of significant correlations for the other markers may simply reflect limited statistical power due to the small sample size, reducing the ability to detect associations even if they exist.

In our study, patients with CLL had increased plasma levels of sCD25, which regulates affinity for IL-2 [31]. Elevated plasma CD25 levels in patients with CLL suggest an activated state of malignant cells, as the increased CD25 expression in patients with CLL is associated with more aggressive disease characteristics, such as a rapid disease progression, and a poorer prognosis [32]. It is worth noting that CD25 expression in patients with CLL differs among patients, and not all patients with CLL exhibit high levels of CD25 [33]. Therefore, assessing CD25 levels in combination with other prognostic markers may provide a more comprehensive understanding of the disease status and risk stratification of patients.

In our study we found increased levels of plasma PD-1 and PD-L1 in patients with CLL. An altered PD-1/PD-L1 axis contributes towards T cell exhaustion in patients with CLL [34]. While PD-1 serves as a marker for T cell exhaustion, the sole blockade of PD-1 has not yielded significant clinical benefits in patients with CLL [35]. Hence, there is a need to identify a more potent combination of therapies for these patients and to stratify them based on the likelihood of benefiting from immune checkpoint therapy. However, our findings, similar to those previously reported [15,36], have several limitations, including a low sample size. This limits the implementation of statistical models and subgroup analysis of patients based on clinical measures such as staging and CLL-IPI score.

The inclusion of African patients with CLL is a key strength of this study, as CLL remains understudied in African populations where biological variation may influence disease behavior. A recent study, for example, showed a higher prevalence of del(17p13) in African patients [13], underscoring the need for population-specific studies. Our findings therefore provide valuable preliminary data on immune checkpoint profiles in this population group and highlight the importance of larger studies to validate these observations.

Future studies need to include larger cohorts with broader clinical and prognostic characterization (including IGHV mutation status and ZAP-70 levels). This will allow for more refined subgroup analyses and a clearer understanding of the biological and prognostic relevance of soluble immune checkpoints in CLL. One of the key limitations of the present study is that we employed a 6-plex soluble immune checkpoint panel, which did not include additional relevant markers such as soluble CD86. Recent evidence suggests that CD86 may have prognostic value in CLL, particularly in predicting time to first treatment and potential resistance to targeted therapies such as ibrutinib and venetoclax [37,38]. Due to these emerging associations, future studies should utilize a broader 12-plex LEGENDplex panel to enable a broader assessment of soluble immune-regulatory molecules. Moreover, the relatively small sample size, particularly within cytogenetic subgroups such as del(11q), del(17p), trisomy 12, and del(13q) limits the statistical power of the subgroup analyses and necessitates cautious interpretation of these findings. Although the total sample size met the minimum requirement identified in the a priori power calculation, the study was not sufficiently powered to conduct definitive subgroup analyses. Consequently, the associations observed between soluble immune checkpoint levels and individual cytogenetic abnormalities should be considered exploratory. Larger studies with broader representation across cytogenetic risk groups are needed to validate these findings and to determine whether the soluble immune checkpoint signatures observed here are consistent in more heterogeneous CLL populations. Additionally, the single-center nature design of this study may introduce potential selection bias, which could limit the generalizability of our findings.

## 4. Methods and Materials

### 4.1. Patients Recruitment

Participants were recruited from King Edward VIII Hospital (KEH), which is a tertiary healthcare facility situated in Durban, KwaZulu-Natal, South Africa. Ethical approval for this study was obtained from the University of KwaZulu-Natal Biomedical Research Ethics Committee (BE456/18), South Africa, on the 14 October 2021. All study participants provided written informed consent as previously described [23]. The recruitment information sheet outlining the study objectives was distributed to patients with CLL attending the hospital, as well as to healthy individuals accompanying them. Interested participants were screened according to the inclusion and exclusion criteria. Those who met the eligibility criteria were provided with an informed consent form to review and sign if they agreed to participate (Figure S1).

### 4.2. Inclusion and Exclusion Criteria

We included untreated patients with CLL diagnosed according to the International Workshop on Chronic Lymphocytic Leukemia (iwCLL) criteria [39], along with age-matched healthy controls with no clinical signs of infection. For the control group, eligible individuals were required to be adults with no personal history of hematological malignancy, autoimmune disease, acute infection, or chronic inflammatory conditions. Controls were also excluded if they were taking immunosuppressive medication or presented with symptoms suggestive of an underlying immune disorder. The included patients were aged 18 years or older.

Individuals diagnosed with other forms of cancer not limited to CLL, as well as those receiving any form of treatment, were excluded. Patients with CLL that have previously received treatment were also excluded. In addition, pregnant or breastfeeding individuals and those with a history of severe allergic reactions or hypersensitivity to blood collection procedures were excluded.

### 4.3. Sample Size Estimation

We calculated the minimum sample size required to detect a large effect size in galectin-9 levels between patients with CLL and healthy controls. Assuming 95% statistical power and a significance level (α) of 0.05, a two-tailed unpaired t-test indicated that a minimum of ten (*n* = 9) patients with CLL and six (*n* = 5) healthy controls were needed. All sample size estimations were performed using G*Power version 3.1.94 (Universität Düsseldorf, Düsseldorf, Germany).

### 4.4. Sample Collection

**Figure 3:**
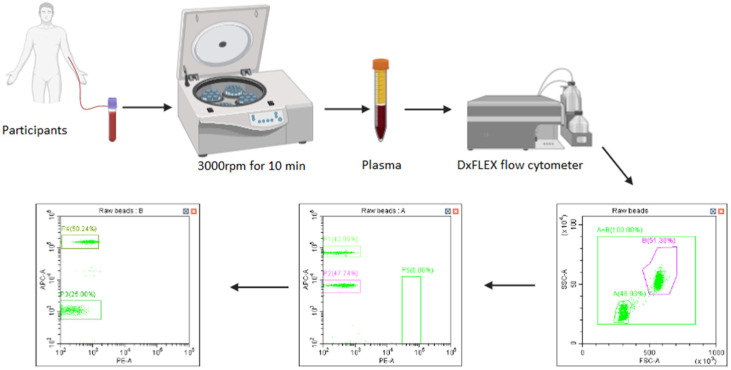
Sample collection. Figure 1 illustrates the sample collection, processing, and sample analysis using flow cytometric bead array-based methods.

Five milliliters (5 mL) of blood was collected into ethylenediamine tetra-acetic acid (EDTA) tubes (BD Bioscience, San Jose, CA, USA) and centrifuged within 1–2 h of collection at 3000 rpm for 10 min at 4 °C (Figure 3). Plasma was then aliquoted and stored at −20 °C for analyses.

### 4.5. Measurement of Soluble Immune Checkpoint Profiles

The soluble immune checkpoints (PD-1, PD-L1, CTLA-4, TIM-3, galectin-9, and sCD25) were measured in plasma using the BioLegend Human Immune Checkpoint Panel 1-S/P LEGENDplex™ kit (BioLegend, San Diego, CA, USA), according to the manufacturer’s instructions. All samples were then transferred into FACS tubes (BD Biosciences, San Jose, CA, USA), and data were acquired using a Beckman Coulter DxFLEX flow cytometer (Beckman coulter, Inc., Brea, CA, USA). The absolute concentration of each analyte was determined using the BioLegend LEGENDplex™ data analysis software based on a standard curve recorded for each analyte.

### 4.6. Measurements of Serum Soluble Beta-2-Microglobulin (B2M) Levels

Plasma levels of beta-2 microglobulin (B2M) were quantified using a human enzyme-linked immunosorbent assay (ELISA) (ThermoFisher Scientific, Waltham, MA, USA), following the manufacturer’s protocol.

### 4.7. Statistical Analysis

The GraphPad Prism version 8 software (GraphPad Software Inc., San Diego, CA, USA) was used to compare data between the two groups. An unpaired Student’s t-test was performed to compare parametric data between the two groups. To correct for multiple comparisons, a Bonferroni-corrected critical *p*-value of <0.01 was considered statistically significant. To investigate the association between soluble immune checkpoints and β2-microglobulin (B2M) levels and Rai stage, we performed multiple linear regression analysis using Python (v3.10) with the statsmodels, SciPy, and seaborn libraries. The model was adjusted for age and sex to account for potential confounding. To investigate the association between soluble immune checkpoints and FISH status as well as CLL-IPI, we performed logistic analysis using STATA 18 [40]. To correct for multiple comparisons, a Bonferroni-corrected critical *p*-value of <0.025 was considered statistically significant.

## 5. Conclusions

Patients with CLL exhibit elevated plasma levels of sCD25, TIM-3, galectin-9, PD-1, and PD-L1. Our findings suggest that galectin-9 may have prognostic value in CLL, as it correlates with an established independent prognostic marker, B2M. However, plasma levels of sCD25, TIM-3, PD-1, and PD-L1 were not associated with prognostic markers such as B2M or chromosomal abnormalities. Therefore, future studies are warranted to validate the significance of these soluble immune checkpoints in larger cohorts of African patients with CLL.

## Figures and Tables

**Table 1:** The baseline characteristics and hematological profiles of the participants.

	Control (*n* = 12)	Patients with CLL [21]	*p*-Value
Gender			
Male, *n* (%)	7 (58.33)	13 (61.9)	
Female, *n* (%)	5 (41.67)	8 (38.1)	
Age (Years)	56.58 ± 15.67	62.33 ± 13.31	0.2714
Hematological parameters			
White blood cell count (10^3^ µL)	5.26 ± 1.38	130.4 ± 29.71	0.0005
Red blood cell (10^6^ µL)	4.74 ± 0.94	2.10 ± 0.84	<0.0001
Hemoglobin (g/dL)	14.13 ± 3.81	8.19 ± 2.30	<0.0001
Platelets (10^3^ µL)	210.4 ± 73.14	157.5 ± 141.9	0.1831
CD38% positive B cells	28.47 ± 19.01	57.39 ± 8.001	0.0002

**Table 2:** Clinical staging and prognostic markers in patients with CLL (*n* = 21).

Clinical Parameters	
RAI Staging	
I, *n* (%)	0 (0)
II, *n* (%)	5 (23.8)
III, *n* (%)	6 (28.6)
IV, *n* (%)	10 (47.6)
FISH Status	
Trisomy 12, *n* (%)	1 (4.8)
Deletions	
11q22, *n* (%)	7 (33.3)
13q14, *n* (%)	6 (28.6)
17p13, *n* (%)	3 (14.3)
no abnormalities, *n* (%)	4 (19.0)
CLL-IPI	
Low risk, *n* (%)	14 (66.7)
Intermediate risk, *n* (%)	4 (19)
High risk, *n* (%)	3 (14.3)
Prognostic Biomarkers	
B2M mg/L	0.74 ± 0.30

## Data Availability

All data supporting the findings of this study are included in the article and its Supplementary Materials. Additional data generated during the study are available from the corresponding author upon request.

## Supplementary Materials

The following supporting information can be downloaded at: https://www.mdpi.com/article/10.3390/ijms27010098/s1.
