# Supplementary material for: Association of Galectin-9 Soluble Immune Checkpoint with Clinical Prognostic Markers in Patients with Chronic Lymphocytic Leukemia
Source: Int J Mol Sci. 2025 Dec 22;27(1):98. doi: 10.3390/ijms27010098 (PMC12785695; doi:10.3390/ijms27010098)
Supplement: Supplementary file 1 [file ijms-27-00098-s001.zip › ijms-4024173-supplementary.pdf]

Supplementary Materials

Table S1: Biological characteristics of patients with CLL

Patient ID	Age	Sex	Rai stage	FISH status	β_2 macroglobulin ((mg/L)
CLL-1	57	F	IV	del(11q22)	0.9
CLL-2	65	M	III	del(13q14)	0.1
CLL-3	68	M	II	del(13q14)	0.8
CLL-4	83	F	IV	del(17p13)	1.0
CLL-5	55	M	III	del(13q14)	0.6
CLL-6	48	M	III	del(13q14)	0.8
CLL-7	67	F	III	del(13q14)	0.6
CLL-8	57	F	IV	del(11q22)	0.5
CLL-9	57	M	II	del(13q14)	0.8
CLL-10	55	M	IV	del(11q22)	0.4
CLL-11	86	F	IV	del(17p13)	0.5
CLL-12	69	F	II	no abnormalities	1.2
CLL-13	64	M	IV	trisomy 12	1.3
CLL-14	77	F	IV	del(17p13)	0.9
CLL-15	54	M	IV	del(11q22)	1.1
CLL-16	64	M	II	no abnormalities	0.5
CLL-17	55	M	III	del(11q22)	0.6
CLL-18	57	M	II	no abnormalities	0.8
CLL-19	27	M	IV	del(11q22)	0.7
CLL-20	61	M	IV	del(11q22)	0.6
CLL-21	83	F	III	no abnormalities	0.8

Table S2: Age, sex adjusted multivariable regression analysis of soluble immune checkpoints with beta-microglobulin 2 (B2M) and Rai stage in patients with CLL

	B2M				Rai stage			
	β (coef)	SE	95% CI	p-value	β (coef)	SE	95% CI	p-value
**sCD25**	-0.048	1.31	–2.61-2.52	0.971	-901.67	768.35	–2406.63-603.29	0.274
**TIM-3**	-3.00	2.13	–7.17-1.17	0.196	-2521.88	1250.58	–4972.97- –70.79	0.078
**Galectin-9**	0.65	0.20	0.26-1.04	**0.012 (*)**	218.93	117.26	–11.89-449.75	0.099
**PD-1**	0.25	0.15	–0.04-0.54	0.123	56.00	86.89	–114.31-226.31	0.537
**PD-L1**	-0.0065	0.018	–0.042-0.029	0.733	2.21	10.79	–18.93-23.35	0.843

Table S3: Odd ratios of soluble immune checkpoints with FISH profiles, International Prognostic Index for Chronic Lymphocytic Leukemia (CLL-IPI) score, in patients with CLL.

Variables	Soluble immune checkpoints	Odds Ratio	95% Confidence Interval	p value
17p13 deletion	TIM-3	1.00	1.00-1.00	0.64
	Galectin-9	0.99	0.97-1.00	0.30
	PD-1	1.00	0.98-1.02	0.77
	PD-L1	0.96	0.79-1.17	0.70
	sCD25	1.00	1.00-1.00	0.88
CLL-IPI	TIM-3	0.99	0.98-1.00	0.27
	Galectin-9	0.96	0.84-1.10	0.54
	PD-1	1.00	0.99-1.00	0.43
	PD-L1	1.00	1.00-1.00	0.38
	sCD25	1.00	1.00-1.00	0.50

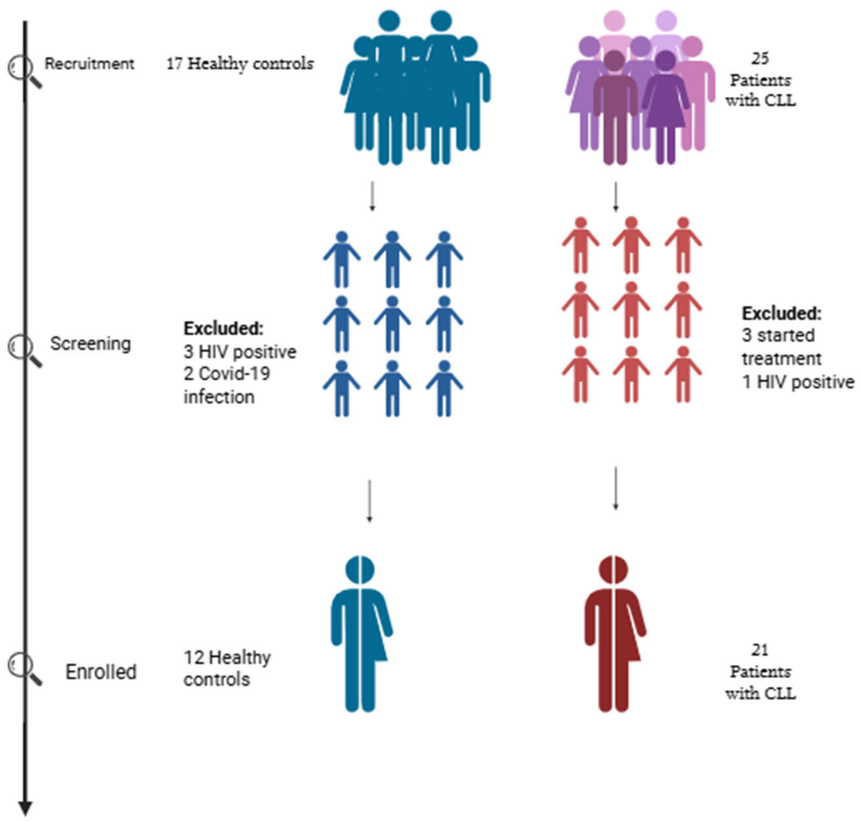

Figure S1: Participant recruitment and enrolment.
